# Associations of TAS1R2 and TAS2R38 Genetic Variants with Sugar-Sweetened Beverage Intake and Obesity Risk in Kuwaiti Adolescents: A Cross-Sectional Study

**DOI:** 10.3390/children12091192

**Published:** 2025-09-08

**Authors:** Razan Yousef, Dalal Usamah Zaid Alkazemi, Mohamed Abu-Farha, Jehad Abubaker, Sriraman Devarajan, Abdur Rahman, Fahd Al-Mulla

**Affiliations:** 1 Department of Food Science and Nutrition, College of Life Sciences, Kuwait University, Shadadiya, Kuwait City 74252, Kuwait; razan.yousef@ku.edu.kw (R.Y.); abdurrahman.ahmed@ku.edu.kw (A.R.); 2 Department of Biochemistry and Molecular Biology, Dasman Diabetes Institute, Kuwait City 15462, Kuwait; mohamed.abufarha@dasmaninstitute.org (M.A.-F.); jehad.abubakr@dasmaninstitute.org (J.A.); sriraman.devarajan@dasmaninstitute.org (S.D.); 3 Department of Translational Research, Dasman Diabetes Institute, Kuwait City 15462, Kuwait; fahd.almulla@dasmaninstitute.org

**Keywords:** taste receptors, *TAS1R2*, *TAS2R38*, sugar-sweetened beverages, obesity, genetic polymorphism, nutrigenetics, adolescents, Kuwait

## Abstract

**Highlights:**

**What are the main findings?**

• The rs10246939 CC genotype of *TAS2R38* was significantly associated with lower sugar-sweetened beverage consumption (*p* = 0.018, OR = 0.24, 95% CI = 0.08–0.79).
• The rs713598 SNP in *TAS2R38* showed a marginal association with BMI percentiles and z-scores, while no significant associations were observed for TAS1R2 SNPs.

**What is the implication of the main finding?**

• Genetic variation in *TAS2R38* may influence dietary preferences and obesity risk in adolescents.
• These results underscore the importance of considering taste receptor polymorphisms in obesity prevention strategies and personalized nutrition approaches.

**Abstract:**

**Background/Objectives**: Obesity is increasing worldwide, driven by unhealthy dietary habits and sedentary lifestyles. Genetic variations in taste receptor genes, particularly *TAS1R2* and *TAS2R38*, may influence taste preferences, dietary intake, and obesity risk. This study examined associations between *TAS1R2* and *TAS2R38* polymorphisms, sugar-sweetened beverage (SSB) intake, and obesity risk in Kuwaiti adolescents. **Methods**: A cross-sectional study was conducted among 260 adolescents aged 11–14 years recruited from public schools in Kuwait. Genotyping of five single-nucleotide polymorphisms (SNPs) was performed using TaqMan assays. Associations between SNPs, SSB intake, and obesity parameters were evaluated using multinomial logistic regression and non-parametric tests, adjusted for age, sex, nationality, BMI z-scores, basal metabolic rate, and physical activity. *p*-values were corrected using the Benjamini–Hochberg method. **Results**: The rs713598 SNP in *TAS2R38* showed a marginal association with BMI percentiles and z-scores. Adolescents carrying the CC genotype of rs10246939 SNP in *TAS2R38* had significantly lower odds of high SSB consumption (>3 servings/week) compared with T-allele carriers (*p* = 0.018, OR= 0.24, 95% CI = 0.08–0.79). No significant associations were detected for *TAS1R2* SNPs. **Conclusions**: Variations in *TAS2R38* were linked to obesity measures and beverage intake in Kuwaiti adolescents, supporting a potential genetic contribution to dietary behaviors. These findings highlight the importance of taste receptor genetics in obesity research, though replication in larger and more diverse populations is required.

## 1. Introduction

Obesity is a significant global health challenge and a major contributor to chronic disease burden. Defined by the World Health Organization (WHO) as excessive fat accumulation impairing health, obesity results from a complex interplay of genetic, metabolic, environmental, and behavioral factors [1]. In 2016, WHO estimated that over one billion people worldwide, including 650 million adults and 340 million children and adolescents aged 5–19 years, were living with obesity [2,3]. Globally, obesity prevalence dramatically increased from 4.6% in 1980 to 14% in 2019 [1]. In Kuwait, childhood overweight and obesity rates increased from 17.7% and 21.4% in 2007 to 20.2% and 28.4% in 2019, respectively [4]. Untreated obesity can lead to chronic inflammation, insulin resistance, mitochondrial dysfunction, oxidative stress, and metabolic disorders such as type 2 diabetes mellitus (T2DM) [5].

Dietary habits significantly impact obesity development. Unhealthy dietary patterns, characterized by frequent high intake of processed foods, refined grains, sugar-sweetened beverages (SSBs), and saturated fats, contribute to excessive caloric intake and weight gain [6,7]. Among these, SSBs are consistently linked with adiposity and metabolic risk [8,9]. The U.S. Dietary Guidelines for Americans (2020–2025) define SSBs as soft drinks, fruit drinks, energy drinks, and sports drinks, while excluding diet beverages, protein-based sports drinks, sweetened teas/coffees, and 100% juices [10]. A cohort study of 1165 children aged 2–18 found that consuming >10% of total energy from sugars was associated with a 2.57-fold increased likelihood of obesity (*p* = 0.002) and 1.77-fold greater odds of overweight (*p* = 0.047) [9]. Supporting this, a recent meta-analysis of 85 studies reported that each additional daily SSB serving was associated with a 0.07 kg/m^2^ higher BMI in children (95% CI: 0.04–0.10), and interventions reducing SSB intake attenuated BMI gain by 0.21 kg/m^2^ (95% CI: −0.40 to −0.01) [11].

Unlike solid sweet foods, SSBs exert distinct metabolic effects because of their rapid digestion, absorption, and metabolism. Their high glycemic index leads to sharp postprandial rises in blood glucose and insulin, elevating the insulin-to-glucagon ratio, which increases hunger and reduces energy expenditure. SSBs also bypass satiety mechanisms, limiting compensatory reductions in subsequent energy intake and producing a net energy surplus. Excessive consumption promotes insulin resistance, inflammation, and T2DM, while high fructose intake contributes to de novo lipogenesis, dyslipidemia, and hepatic insulin resistance due to unregulated hepatic uptake. Importantly, early-life exposure to SSBs fosters a preference for sweet taste [8].

Genetic variation further modulates taste perception, shaping food choices, dietary habits, and ultimately disease risk. These differences may reduce the effectiveness of uniform dietary recommendations and contribute to childhood obesity [12,13,14,15]. For instance, children genetically predisposed to prefer sweet or fatty tastes may favor energy-dense foods while rejecting nutrient-rich but bitter-tasting vegetables such as broccoli or kale [16,17]. Understanding these predispositions could support the development of personalized nutrition strategies that enhance obesity prevention efforts.

Taste perception is mediated by specific genes, notably *TAS1R2* and *TAS2R38*, which influence sweet and bitter preferences, respectively. The *TAS1R2* gene encodes part of the sweet taste receptor, with SNPs rs35874116 and rs9701796 linked to sugar intake and dietary behaviors, though findings are inconsistent across cohorts [18,19,20,21,22]. The *TAS2R38* gene influences bitter perception through common SNPs rs713598, rs1726866, and rs10246939, which define the PAV (supertaster) and AVI (non-taster) haplotypes [23,24]. Evidence from child and infant cohorts suggests associations with higher energy intake from sweet foods [22,25], though other studies report null findings [15,26]. These discrepancies may reflect differences in study design, dietary assessments, and age groups examined.

While genome-wide association studies (GWAS) have identified loci linked to taste and diet [27], candidate gene studies remain essential for hypothesis-driven research, particularly in underrepresented populations. In Kuwait, where obesity prevalence is among the highest globally, no prior studies have investigated *TAS1R2* and *TAS2R38* variants in relation to SSB consumption in adolescents. Given their rapid absorption, metabolic consequences, and lack of satiety compensation, SSBs were selected as the dietary focus of this study. We therefore investigated associations between *TAS1R2* and *TAS2R38* variants, SSB intake, and obesity risk in Kuwaiti adolescents, aiming to contribute to the growing field of nutrigenetics and to inform future personalized nutrition interventions.

## 2. Materials and Methods

This study employed a cross-sectional design based on the sampling framework of a previously established school-based cohort study to determine the associations between genetic variations in *TAS1R2* and *TAS2R38*, obesity risk, and SSB consumption [28]. Participants were randomly recruited from 12 public middle schools across all six governorates of Kuwait to ensure geographic representativeness. Written informed consent from parents and verbal assent from children were obtained, including permission for laboratory testing and anonymous DNA analysis. The study adhered to the Helsinki Declaration, and ethical approvals were granted by Kuwait’s Ministry of Health (No: 2015/248), the Health Sciences Centre Ethics Committee at Kuwait University (No: DR/EC/2338), and Dasman Diabetes Institute’s Ethical Review Committee (RA2017-026).

Initially, 432 children aged 11–14 years were recruited using a stratified multistage cluster random sampling method, proportionally representing each governorate based on population size. Exclusion criteria encompassed children diagnosed with genetic syndromes, chronic medical conditions, or those receiving medications known to induce significant weight fluctuations. A minimum sample size of 310 participants was calculated assuming a childhood obesity prevalence of 28.39% [4], a 95% confidence level, and a 5% margin of error. This ensured sufficient power to detect statistically meaningful differences.

Demographic characteristics, including age, sex, nationality, parental income, and educational level, were collected using self-reported questionnaires completed by parents. Anthropometric measurements, including height, weight, and waist circumference (WC), were performed by trained researchers following standardized protocols. BMI-for-age percentiles (BMI%) and z-scores (BMIz) were calculated based on CDC growth reference data to classify children using PediTools: Fenton 2013 (Accessed 20 October 2024) [29]. The waist-to-height ratio (WHtR) was also determined to evaluate central adiposity.

The estimated energy intake was calculated using the Schofield method based on participants’ actual body weight, as this better reflects metabolic demand in children and adolescents. Although kcal/kg IBW provides a standardized reference, we used actual weight in line with current pediatric dietary guidelines [30,31,32]. Additionally, a validated questionnaire was used to collect data about participants’ physical activity (PA) level. This questionnaire was built based on The Arab Teens Lifestyle Study [30], validated with high school students, and demonstrated a strong association with accelerometer data (Spearman correlation = 0.92, *p* < 0.001, for total step count) [28]. The questionnaire included data on the frequency, duration, and intensity of physical activity across different domains (e.g., sport participation, household chores, transportation). Activities were assigned MET values based on the Youth Compendium of Physical Activities. Participants’ total MET-minutes/week were then calculated and used to classify physical activity as low (<600 MET-minutes/week), medium (600–3000 MET-minutes/week), or high (>3000 MET-minutes/week) [33].

SSB intake was assessed using a single-item frequency question administered in person by dietitians trained in dietary data collection for adolescents about their consumption of SSB over the last 12 months. Although this question was part of a previously validated FFQ for Middle Eastern children [34], for the present study, we analyzed only the sugar-sweetened soft drink item, which excluded diet/no-sugar soft drinks. Other beverages such as milk, 100% fruit juices, and unsweetened teas or coffees were not classified as SSBs, and were therefore excluded, consistent with the definitions outlined in the U.S. Dietary Guidelines for Americans 2020–2025 [35], which classify SSBs as soft drinks, fruit drinks, energy drinks, and sports drinks, but exclude diet/no-sugar soft drinks, milk, 100% fruit juices, and unsweetened tea or coffee. The original FFQ correlated (r = 0.72) with total sugar intake compared to 3-day food records. Following approaches used in prior studies on adolescent SSB intake [36,37], participants were categorized into three groups: low consumers (<1 time/week), medium consumers (2–3 times/week), and high consumers (>3 times/week). This classification allowed comparability with published research and captured meaningful intake differences across the sample.

Blood samples were collected and stored between February and April 2016. Blood glucose levels were analyzed to validate SSB consumption. Moreover, DNA extraction, sequencing, and genotyping were subsequently performed at the laboratories of the Dasman Diabetes Institute. Venous blood samples were collected without prior fasting by trained school nurses using EDTA tubes. DNA extraction utilized the Gentra Puregene Kit (Qiagen, Valencia, CA, USA), with DNA quantified using the Quant-iT PicoGreen dsDNA Assay Kit (Life Technologies, Grand Island, NY, USA). DNA purity and concentration were verified using the Epoch Microplate Spectrophotometer (BioTek Instruments, Winooski, VT, USA). Absorbance values were examined at 260–280 nm to ensure they fell within an optical density range of 1.8–2.1.

DNA amplification was performed via polymerase chain reaction (PCR) with specifically designed forward and reverse primers. Candidate SNP genotyping employed Taq-Man Genotyping Assays on the ABI 7500 Real-Time PCR System (Applied Biosystems, Foster City, CA, USA). These candidate SNPs were chosen based on previous evidence of relevance in taste perception and dietary intake [16,17,19,20,21,22,25]. Each reaction included 10 ng DNA, 5× FIREPOL Master Mix (Solis Bio Dyne, Tartu, Estonia), and 1 µL 20× TaqMan SNP Genotyping Assay. Cycling conditions were 60 °C for 1 min, 95 °C for 15 min, and 40 cycles at 95 °C for 15 s and 60 °C for 1 min. Sanger sequencing results were used to validate genotyping accuracy.

Data analysis was performed using SPSS software (version 26, IBM Corp., Armonk, NY, USA). Descriptive statistics summarize demographic characteristics and genotype frequencies. The Chi-squared test compared observed and expected genotype frequencies calculated by the Hardy–Weinberg equilibrium. The Benjamini–Hochberg correction method was applied to control for type 1 error after multiple comparisons and was set at a false discovery rate (FDR) of 0.10. Chi-square tests evaluated genotype-weight status associations, and Mann–Whitney tests compared SNP genotypes to adiposity parameters. Normality was assessed with the Kolmogorov–Smirnov test. Associations between SNPs and SSB consumption were analyzed through multinomial logistic regression models adjusted for sex, nationality, BMIz scores, BMR, and PA. Statistical significance was set at *p* < 0.05. Given sparse genotype cells in some models, estimates are interpreted with caution, focusing on effect sizes and 95% CIs. Future multi-center studies and IPD meta-analyses are planned to improve precision and assess heterogeneity across populations. Post hoc power analysis was performed using the G*Power software (Version 3.1.9.7, Accessed 14 April 2025) to check the achieved power of the performed statistical tests. Multiple imputation was performed to handle missing data.

## 3. Results

**Figure 1:**
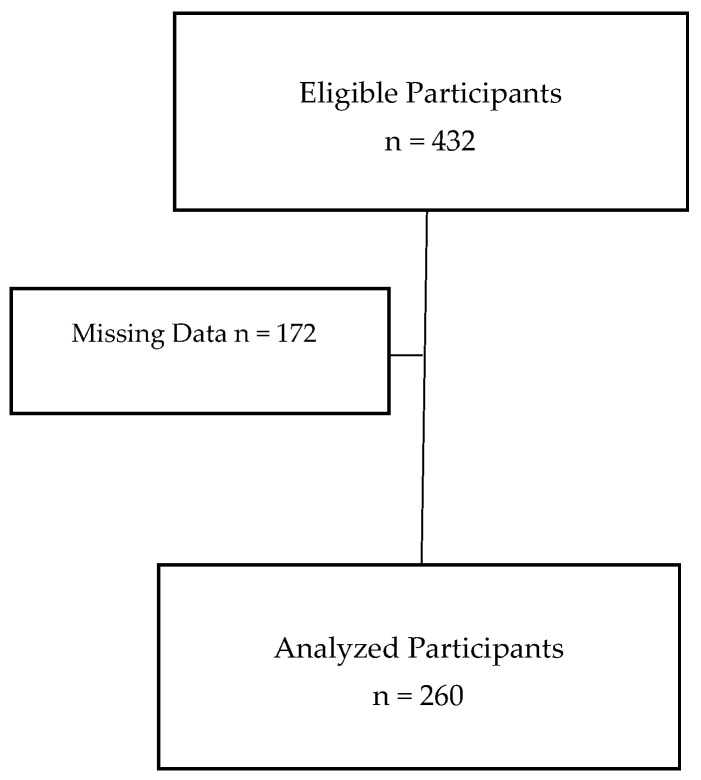
Exclusion of participants with no anthropometric, dietary, or genotype data.

From an initial sample of 432 recruited children, 172 participants were excluded due to incomplete dietary, anthropometric, and genotypic information. This resulted in a final analytical sample of 260 participants (see Figure 1).

### 3.1. Demographic and Socioeconomic Characteristics

Table 1 presents the demographic and socioeconomic characteristics of the study participants, which included 113 boys and 147 girls. There was no significant difference in mean age between boys (12.01 ± 0.85 years) and girls (11.84 ± 0.90 years, *p* = 0.133). However, nationality distribution differed significantly, with a greater proportion of girls being Kuwaiti than boys (86.4% vs. 52.2%, *p* < 0.001). In terms of geographic distribution, girls were predominantly concentrated in Al-Ahmadi Governorate (47.3%), while boys were more evenly distributed, particularly across Al-Ahmadi (27.4%) and Al-Jahra (24.8%) (*p* < 0.001). There were no significant gender differences in socioeconomic indicators, parental smoking status, weight classification, or SSB consumption.

### 3.2. Genotypic and Allelic Frequencies

Table 2 presents the genotypic and allelic frequencies for five SNPs located in the *TAS1R2* and *TAS2R38* genes among the study participants. All genotypic distributions closely adhered to Hardy–Weinberg expectations, confirming genetic consistency. This step validated the representativeness of the genotypic distribution. A sex-nationality-and socioeconomic-stratified analysis showed no statistically significant difference between wild and mutant allele carriers.

### 3.3. Estimated Dietary Intake and Physical Activity Level, and Validation of SSB Consumption with Blood Glucose Levels

Table 3 shows the estimated dietary intake and physical activity level of participants based on their genotypes. There were no statistically significant differences among participants across different genotypes in their estimated dietary intake and physical activity level. Table 4 shows the validation of SSB with blood glucose levels by performing a comparative analysis of the mean blood glucose levels across the SSB consumption categories using the Kruskal–Wallis’s test. The results showed no statistically significant differences in the mean blood glucose levels across SSB consumption categories (*p >* 0.05).

### 3.4. Anthropometric Measurements and Genotypic Associations

Table 5 presents the comparisons between genetic variations in *TAS1R2* and *TAS2R38* in key anthropometric parameters. Among the five SNPs analyzed, marginally significant differences in BMI percentile and BMI z-scores were observed for rs713598. Adolescents with the CC genotype of rs713598 had higher BMI% (77.49% vs. 68.59%, *p* = 0.050) and BMIz (1.12 vs. 0.76, *p* = 0.050) compared to those carrying GC/GG genotypes. Although the initial Mann–Whitney U test yielded *p*-values of 0.05 for both BMI% and BMIz, these comparisons reached statistical significance following multiple imputations, indicating potential bias in the complete-case analysis.

### 3.5. SSB Consumption and Genotypic Associations

**Figure 2:**
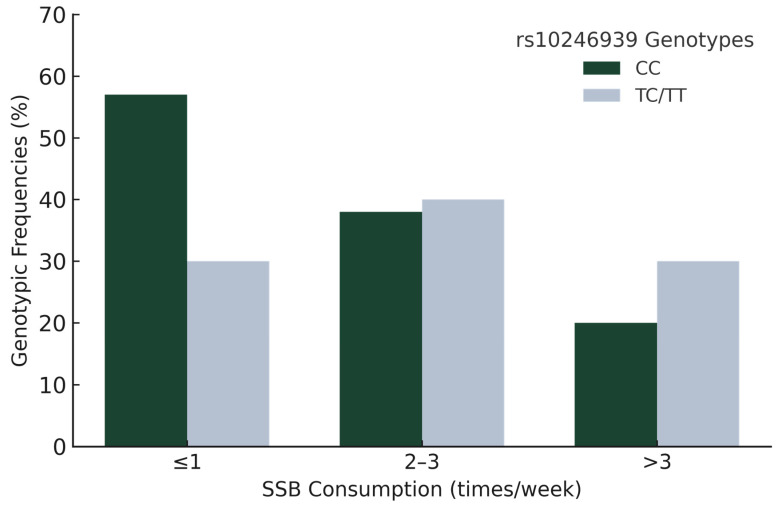
CC genotype carriers were 76% less likely to consume higher SSBs (>3 servings/week) compared to those carrying the T allele, indicating a potential genetic influence on SSB consumption behavior (*p* = 0.018). Genotypic frequencies based on SSB consumption/week.

Table 6 presents the results of a multinomial logistic regression analysis exploring the associations between genetic variants and SSB consumption. Among the SNPs analyzed, rs10246939 emerged as the only variant significantly associated with SSB intake (*p* = 0.018), even after adjusting for age, sex, nationality, BMIz scores, estimated BMR, and PA level, and by correcting for multiple comparisons using the Benjamini–Hochberg correction method with an FDR set at 0.10 (adjusted *p*-value *=* 0.090 < FDR = 0.10). Notably, the odds that adolescents would carry the CC genotype were 76% (OR = 0.24, 95% CI = (0.08–0.79)) less likely to consume higher SSBs (>3 servings/week) compared to those carrying the T allele (*p* = 0.018) (Figure 2). The association observed in the multinomial logistic regression was statistically significant in the initial analysis (*p*-value = 0.018), but this effect attenuated after multiple imputations (*p*-value = 0.049), suggesting that missing data may have influenced the strength of the association. In contrast, no significant associations were observed for the other SNPs analyzed. Post hoc power analysis indicated 100% power to detect the observed odds ratio (0.24) at α = 0.05 with a sample size of 260. This indicates that the study was powered to detect a large effect at an alpha level of 0.05. Additionally, a Kruskal–Wallis H test showed no statistically significant difference in BMIz score across the SSB consumption categories, H = 4.113, *p* = 0.128.

## 4. Discussion

This study explored the associations between genetic variants in taste receptor genes, specifically *TAS1R2* and *TAS2R38*, with obesity risk and SSB consumption among Kuwaiti adolescents. Our key findings indicate that variations in the bitter taste receptor gene *TAS2R38* were nearly associated with obesity parameters, such as BMI% and BMIz, and significantly with the frequency of SSB consumption.

Specifically, adolescents who carried the wild genotype of rs713598 SNP in *TAS2R38* had higher BMI% and BMIz compared to those carrying the mutant allele. These findings suggest that *TAS2R38* variants may be associated with higher adiposity, either directly or through dietary preferences. Given the genetic basis of these variants, their influence on obesity development may be biologically plausible. Our results align well with previous research by Feeney et al., who similarly reported that children with the *TAS2R38* PAV haplotype, classified as “supertasters,” tended to have higher body weights [16]. In contrast, our findings diverge from studies by Wang et al. and O’Brien et al., which found no significant relationship between *TAS2R38* genotype and adiposity [26,38]. These discrepancies in the literature highlight the complex and subtle relationship between taste genetics and obesity, reinforcing the notion that individual genetic variants in taste receptor genes might not consistently or strongly influence obesity markers in isolation.

No associations were observed between *TAS1R2* polymorphisms and obesity parameters in our sample. However, some previous studies have reported significant relationships. For example, Pioltine et al. observed that the wild type of the *TAS1R2* SNP rs9701796 was associated with an increased WHtR among adolescents [21]. Variations in findings between studies might be attributed to differences in age groups, dietary environments, sample sizes, or statistical power. It is plausible that genetic variations in bitter taste perception might affect children’s dietary preferences, such as aversion toward certain bitter vegetables or increased preference for sweet flavors, potentially leading to higher caloric intake and subsequent adiposity. Given that genotypes are determined at birth and generally remain independent of external lifestyle factors, the observed result in our study supports the hypothesis that *TAS2R38* variants might exert a meaningful influence on adolescent obesity development.

In addition to associations with obesity parameters, our study evaluated relationships between genetic variants and dietary behaviors, specifically SSB consumption. The SSB consumption was validated with blood glucose levels. The results showed no statistical significance (*p*-value = 0.969). It is noteworthy that the mean glucose level in those who consume > 3 times/week was the highest and reached the lower borderline of prediabetes (5.6 mmol/L) according to American Diabetes Association (ADA) diagnostic criteria [39]. Despite the nonsignificant results, this trend may warrant further investigation into larger samples.

We identified a significant association between the *TAS2R38* SNP rs10246939 and the frequency of SSB intake. Adolescents carrying the CC genotype of this variant consumed SSBs less frequently than those with the mutant genotype (*p* = 0.018, OR = 0.24, 95% CI = (0.08–0.79)). This result remained significant after adjusting for covariates (age, sex, nationality, BMIz score, BMR, and PA) and following correction for type I error. None of the other investigated SNPs demonstrated a significant relationship with SSB consumption in our sample.

The existing literature on taste receptor genetics and dietary habits provides mixed evidence. Our findings align with studies conducted in diverse cultural contexts and age groups. For example, Feeney and O’Brien (Ireland, 7–13 years) [15] and Pioltine (Brazil, 7–18 years) [20] reported associations between *TAS2R38* variants and sweet preference in populations where SSBs are commonly consumed but influenced by different dietary norms. Similarly, Keller (United States, 4–6 years) [16] and Chamoun (Canada, 1.5–5 years) [18] examined younger cohorts in Western settings where early-life exposure to SSBs is frequent, often beginning in preschool. These cultural contrasts suggest that the influence of *TAS2R38* polymorphisms on dietary behaviors may be shaped by both age and the surrounding food environment. Our study extends this evidence to Middle Eastern adolescents, where SSBs are widely available and socially embedded, highlighting the importance of considering cultural as well as biological determinants of dietary behavior.

Nevertheless, some studies have reported contrasting results. Keller et al. and O’Brien et al. observed no significant dietary differences by *TAS2R38* genotype [16,25], while others found genotype-dependent associations with *TAS1R2*. For instance, Chamoun et al. showed that preschool children carrying the TT genotype of *TAS1R2* SNP rs35874116 consumed a higher proportion of their calories from sugars, particularly through sweet snacks [18]. Similarly, Pioltine et al. reported greater chocolate powder intake among adolescents with the same genotype [20].

Certain variations in *TAS2R38* (e.g., rs713598) have been associated with differing dietary patterns, including snack and total energy consumption in some populations. These varied findings underscore that relationships between taste receptor polymorphisms and dietary intake are subtle, complex, and context-dependent. Factors such as participant age, cultural dietary norms, and differences in dietary assessment methods (e.g., FFQ versus 24-h dietary recalls) might influence the detection and strength of these associations. Our observed link between rs10246939 (a *TAS2R38* variant) and SSB intake is intriguing but contradicts some prior research; as such, it should be viewed as a hypothesis-generating result. Further studies are needed to confirm this potential relationship under different conditions and in larger samples.

The potential mechanisms linking *TAS2R38* polymorphisms to obesity risk may involve both gustatory and non-gustatory pathways. Altered bitter taste perception can lead to avoidance of nutrient-rich foods containing bitter compounds and compensatory consumption of energy-dense, palatable foods, resulting in higher caloric intake. Beyond taste perception, bitter taste receptors also play roles in regulating satiety and hunger hormones (e.g., glucagon-like peptide-1 [GLP-1], cholecystokinin [CCK]), glucose homeostasis, and gut motility. Variants in *TAS2R38* may therefore influence appetite regulation and energy balance through both behavioral and physiological pathways, ultimately contributing to adiposity [40].

While genetic variations in taste receptor genes influence dietary preferences, other critical factors significantly shape children’s food choices and preferences by inducing epigenetic modifications like DNA methylation or histone modification, which eventually alter gene expression without changing the DNA sequence. In addition to that, early childhood exposure to diverse foods is essential in developing lifelong eating habits. For instance, repeated exposure to various flavors, especially during the complementary feeding period, promotes acceptance and preference for fruits and vegetables. Initially rejected bitter flavors typically become acceptable after multiple exposures [41]. Moreover, dietary preferences and eating behaviors are influenced by complex neural mechanisms associated with appetite regulation. Internal physiological states, such as hunger and satiety, modulate taste perception and food preferences. Fu et al. reported increased preferences for sweet tastes in response to hunger, although the exact neural mechanisms underlying such taste modifications remain unclear [42]. This complexity suggests that genetic influences interact closely with environmental and physiological factors to determine overall dietary preferences and behaviors.

To our knowledge, this is the first study to investigate associations between *TAS1R2* and *TAS2R38* polymorphisms and SSB intake in Kuwaiti adolescents. In addition, we identified genotypic and allelic frequencies for these sweet (*TAS1R2:* rs35874116, rs9701796) and bitter (*TAS2R38*: rs713598, rs1726866, rs10246939) taste receptor SNPs using the gold-standard method of Sanger sequencing. Rigorous quality control procedures—including optimal DNA concentration, primer design, and reagent storage—ensured accurate genotyping. Furthermore, anthropometric risk was assessed using both BMI z-scores and central adiposity indicators (WC and WHtR), offering a more nuanced understanding of obesity risk.

While our findings suggest a link between *TAS2R38* variants and SSB intake, the cross-sectional design precludes causal inference. Stronger validation could be achieved through prospective dietary tracking combined with functional taste perception assessments, such as quantitative sensory testing (QST). Incorporating these approaches in future research would allow for a more robust evaluation of genotype-driven dietary preferences and help clarify the behavioral and physiological mechanisms underlying these associations.

Despite valuable insights, our study has several limitations. First, SSB intake was assessed using a single item from a previously validated FFQ that has not been specifically validated in Kuwaiti youth. This may have introduced recall bias or misclassification, particularly among children or their parents, and could have diluted or underestimated true associations between genetic variants and dietary behaviors. Future studies would benefit from using more comprehensive dietary assessments or validation against objective biomarkers of sugar intake. To partially address this limitation, we compared mean blood glucose levels across SSB consumption categories. Although differences were not statistically significant (*p* = 0.969), mean glucose levels were highest among those consuming SSBs >3 times/week and approached the ADA prediabetes threshold. While exploratory, this finding highlights the potential value of biomarker validation for improving interpretability and generalizability in future studies. Second, only SSB intake was measured, preventing evaluation of overall dietary patterns. A complete FFQ would have provided broader insights into total energy intake and diet quality. Third, small subgroup sizes for certain genotypes reduced statistical power, particularly among adolescents homozygous for minor alleles, raising the risk of type II errors. To strengthen robustness, future work should pool data across similar regional cohorts using harmonized protocols and, where possible, conduct individual-participant-data meta-analyses to increase precision, examine between-study heterogeneity, and validate the direction and magnitude of associations. In line with best practice, our interpretation prioritizes effect sizes and confidence intervals over sole reliance on *p*-values. Our final sample of 260 participants also fell below the calculated minimum, further limiting subgroup analyses and suggesting that significant findings, such as those for TAS2R38, should be interpreted with caution until replicated in larger cohorts. Finally, residual confounding from population stratification and unmeasured lifestyle factors cannot be excluded. Although Kuwaiti and non-Kuwaiti participants were analyzed together, differences in nationality, geography, or allele frequencies may have influenced results. Additional unmeasured factors, including detailed physical activity, broader dietary quality, and socioeconomic status, could also act as confounders or mediators. The absence of these data represents another important limitation.

While GWAS are considered the gold standard for identifying novel genetic associations, hypothesis-driven candidate gene studies continue to play a critical role in evaluating specific variants with established physiological functions. Our focus on *TAS1R2* and *TAS2R38* provides insight into culturally relevant dietary behaviors and the heightened obesity risk among adolescents in Kuwait, a region that remains underrepresented in nutrigenomics research. Although no significant differences in genotypic distributions were observed between Kuwaiti and non-Kuwaiti participants or by socioeconomic status, residual confounding from unmeasured cultural, genetic, and lifestyle factors cannot be excluded. This underscores the need for larger and more diverse cohorts to validate our findings.

Future research should prioritize pediatric populations with broader ethnic representation, employ validated dietary assessment tools tailored to local dietary patterns, and apply rigorous controls for potential confounders (e.g., stratified analyses by ethnicity). Expanding the genetic scope beyond *TAS1R2* and *TAS2R38* to include other taste-related genes would also provide a more comprehensive picture of the genetic influences on dietary behaviors. For example, CD36 is involved in fat taste perception and lipid preference, *TAS1R2/TAS1R3* contribute to umami perception and interact with sweet and bitter signaling pathways, while *TRPM5* plays a central role in transducing sweet, bitter, and umami tastes and modulating subsequent hormonal responses that regulate nutrient intake [43]. A deeper understanding of these pathways could facilitate the design of personalized nutrition strategies and targeted obesity-prevention interventions tailored to children’s genetic taste profiles.

## 5. Conclusions

Overall, this study offers new evidence that genetic differences, especially in the bitter taste receptor gene *TAS2R38* are potentially associated with BMI and SSB intake, as the subgroups were too small to draw strong conclusions. These results are in line with the idea that bitter taste genetics may contribute to pediatric obesity. However, associations between taste-related genes and eating behaviors remain subtle and inconsistent across the literature. Given limitations such as the simplistic dietary assessment, small subgroup sizes, and the possibility of residual confounding, these findings should be viewed with caution.

## Figures and Tables

**Table 1:** Participant’s characteristics.

Characteristics		Boys (*n* = 113)	Girls (*n* = 147)	Total (*n* = 260)	*p*-Value	Adjusted *p*-Value
Age (yrs)	Mean ± SD	12.01 ± 0.85	11.84 ± 0.90	11.92 ± 0.88	0.133 ^1^	0.266
		*n* (%)	*n* (%)	*n* (%)		
Nationality	Kuwaiti	59 (52.20)	127 (86.40)	186 (71.50)	<0.001 ^2^	0.005
	Non-Kuwaiti	54 (47.80)	20 (13.60)	74 (28.50)		
Government of Residence	Capital	17 (15.00)	14 (9.60)	31 (12.00)	<0.001 ^2^	0.005
	Hawally	23 (20.40)	7 (4.80)	30 (11.60)		
	Al-Farwaniyah	10 (8.80)	2 (1.40)	12 (4.60)		
	Al-Jahra	28 (24.80)	28 (19.20)	56 (21.60)		
	Mubarak Al-Kabeer	4 (3.50)	26 (17.80)	30 (11.60)		
	Al-Ahmadi	31 (27.40)	69 (47.30)	100 (38.60)		
Father Education	Secondary school or lower	45 (39.80)	67 (46.50)	112 (43.60)	0.065 ^2^	0.217
	Diploma	18 (15.90)	33 (22.90)	51 (19.80)		
	College degree or higher	50 (44.20)	44 (30.60)	94 (36.60)		
Mother Education	Secondary school or lower	40 (35.40)	56 (38.40)	96 (37.10)	0.884 ^2^	0.887
	Diploma	24 (21.20)	29 (19.90)	53 (20.50)		
	College degree or higher	49 (43.40)	61 (41.80)	110 (42.50)		
Father Income	<=1000 KD	31 (27.70)	50 (34.70)	81 (31.60)	0.558 ^2^	0.698
	1001–1500 KD	39 (34.80)	38 (26.40)	77 (30.10)		
	1501–2000 KD	15 (13.40)	22 (15.30)	37 (14.50)		
	>2000	13 (11.60)	19 (13.20)	32 (12.50)		
	Do Not Know	14 (12.50)	15 (10.40)	29 (11.30)		
Mother Income	<=1000 KD	48 (57.80)	68 (55.70)	116 (56.60)	0.887 ^2^	0.887
	1001–1500 KD	12 (14.50)	22 (18.00)	34 (16.60)		
	1501–2000 KD	7 (8.40)	13 (10.70)	20 (9.80)		
	>2000 KD	4 (4.80)	4 (3.30)	8 (3.90)		
	Do Not Know	12 (14.50)	15 (12.30)	27 (13.20)		
Weight Status	Not Overweight	53 (46.90)	76 (51.70)	129 (49.60)	0.261 ^2^	0.435
	Overweight/Obese	60 (53.10)	71 (48.30)	131 (50.40)		
SSB Consumption	≤1	32 (28.30)	53 (36.60)	85 (32.90)	0.118 ^2^	0.266
	2–3	40 (35.40)	56 (38.60)	96 (37.20)		
	>3	41 (36.30)	36 (24.80)	77 (29.80)		
Parents Smoking Status	No	72 (63.70)	97 (66.00)	169 (65.00)	0.401 ^2^	0.573
	Yes	41 (36.30)	50 (34.00)	91 (35.00)		

^1^ An independent sample *t*-test was used to determine if there is a statistical difference between sexes in their age. ^2^ Pearson’s Chi-squared test was used to determine if there is a statistical difference in these variables between sexes. Benjamini–Hochberg correction method (FDR = 0.01) was applied to adjust the *p*-values. Yrs: years, SD: standard deviation, SSB: sugar-sweetened beverages.

**Table 2:** Genotypic and allelic frequencies of participants.

Gene	SNP	Genotype	*n* (%)	Expected Frequency *n* (%)	*p*-Value	Allele Frequency	
*TAS1R2*	rs35874116	CC	25 (10.00)	25.3 (10.12)	0.997	C	0.318
		TC	109 (43.60)	108.4 (43.36)		T	0.682
		TT	116 (46.40)	116.3 (46.52)			
		Total	250 (100.00)	250 (100.00)			
	rs9701796	CC	161 (66.30)	166.5 (68.52)	0.061	C	0.828
		GC	80 (32.90)	69.3 (28.52)		G	0.172
		GG	2 (0.80)	7.2 (2.96)			
		Total	243 (100.00)	243 (100.00)			
*TAS2R38*	rs713598	CC	99 (39.00)	100.1 (39.41)	0.953	C	0.628
		GC	121 (47.60)	118.7 (46.73)		G	0.372
		GG	34 (13.40)	35.2 (13.86)			
		Total	254 (100.00)	254 (100.00)			
	rs1726866	AA	38 (15.10)	40.4 (16.03)	0.809	A	0.401
		GA	126 (50.00)	121.0 (48.02)		G	0.599
		GG	88 (34.90)	90.6 (35.95)			
		Total	252 (100.00)	252 (100.00)			
	rs10246939	CC	33 (13.40)	37.5 (15.24)	0.490	C	0.390
		TC	126 (51.20)	117.1 (47.60)		T	0.610
		TT	87 (35.40)	91.4 (37.16)			
		Total	246 (100.00)	246 (100.00)			

Expected frequencies were calculated using Hardy–Weinberg equilibrium equation. Chi-squared test was used to determine if there was a statistically significant difference between observed and expected frequencies. SNP: single-nucleotide polymorphism. *TAS1R2*: taste receptor, type1, member 2. *TAS2R38*: taste receptor, type2, member 38.

**Table 3:** Estimated basal metabolic rate (BMR) and physical activity level across genotypes.

Gene	Genotype	Diet	*p*-Value	Physical Activity			*p*-Value
		BMR (Kcal/Day)		*n* (%)			
				Low	Medium	High	
*TAS1R2*	rs35874116		0.577				0.309
	CC	1477.93		12 (48.00)	5 (20.00)	8 (32.00)	
	TC/TT	1457.93		79 (35.10)	76 (33.80)	70 (31.10)	
	rs971796		0.705				0.922
	CC	1449.14		58 (36.00)	52 (32.30)	51 (31.70)	
	GC/GG	1469.23		30 (36.60)	28 (34.10)	24 (29.30)	
*TAS2R38*	rs713598		0.186				0.307
	CC	1473.57		36 (36.40)	37 (37.40)	26 (26.30)	
	GC/GG	1451.77		58 (37.40)	45 (29.00)	52 (33.50)	
	rs1726866		0.293				0.468
	AA	1473.02		30 (34.10)	33 (37.50)	25 (28.40)	
	GA/GG	1452.03		63 (38.40)	49 (29.90)	52 (31.70)	
	rs10246939		0.963				0.284
	CC	1449.98		14 (42.40)	7 (21.20)	12 (36.40)	
	TC/TT	1460.54		74 (34.70)	75 (35.20)	64 (30.00)	

Kruskal–Wallis H test results for mean differences of BMR across genotypes and Chi-square results for differences of PA levels across genotypes. *TAS1R2:* taste receptor, type1, member 2. *TAS2R38:* taste receptor, type2, member 38. BMR values were calculated using the Schofield method and do not represent actual dietary intake. Thus, calories from SSBs or other foods are not included.

**Table 4:** Validation of SSB consumption with blood glucose levels.

SSB Consumption/Week	*n* (%)	BG		*p*-Value
		Mean (mmol/L)	Standard Deviation	
≤1 times	85 (33.20)	5.06	1.18	0.969
2–3 times	95 (37.11)	4.96	0.75	
>3 times	76 (29.69)	5.59	4.29	
Total	256 (100.00)	5.18	2.48	

Kruskal–Wallis’s H Test was used to compare means of blood glucose with consumption of SSB/week. SSB: sugar-sweetened beverage, BG: blood glucose, mmol/L: millimole/liter.

**Table 5:** Mean differences in anthropometric parameters across genotypic variations.

Gene	SNP	Genotype	Mean ± SD	95% CI	Median [25th–75th]	*p*-Value
	BMI%					
*TAS1R2*	rs35874116	CC	72.93 ± 30.48	(60.35, 85.51)	87.40 [47.35–97.38]	0.686
		TC/TT	71.40± 29.41	(67.65, 75.38)	83.40 [52.10–95.62]	
	rs9701796	CC	70.33 ± 30.39	(65.60, 75.06)	83.60 [45.55–95.46]	0.479
		GC/GG	74.10 ± 27.63	(68.02, 80.17)	85.05 [61.73–96.32]	
*TAS2R38*	rs713598	CC	77.49 ± 24.30	(72.64, 82.33)	87.70 [66.80–96.72]	0.050
		GC/GG	68.59 ± 31.79	(63.54, 73.63)	82.80 [43.60–95.70]	
	rs1726866	AA	76.48 ± 24.98	(71.18, 81.77)	87.55 [63.18–97.07]	0.089
		GA/GG	69.33 ± 31.21	(64.51, 74.14)	83.20 [45.53–95.43]	
	rs10246939	CC	73.90 ± 29.83	(63.32, 84.48)	85.10 [63.40–96.70]	0.577
		TC/TT	71.92 ± 28.97	(68.01, 75.84)	83.90 [52.35–96.03]	
	BMIz					
*TAS1R2*	rs35874116	CC	0.92 ±1.18	(0.43, 1.40)	1.15 [−0.07–1.94]	0.690
		TC/TT	0.88 ± 1.23	(0.72, 1.04)	0.97 [0.05–1.72]	
	rs9701796	CC	0.84 ± 1.25	(0.64, 1.03)	0.98 [−0.11–1.69]	0.482
		GC/GG	0.97 ± 1.17	(0.71, 1.22)	1.04 [0.30–1.79]	
*TAS2R38*	rs713598	CC	1.12 ± 1.09	(0.90, 1.34)	1.16 [−0.43–1.84]	0.050
		GC/GG	0.76 ± 1.28	(0.56, 0.96)	0.95 [−0.16–1.72]	
	rs1726866	AA	1.09 ± 1.13	(0.86, 1.34)	1.16 [0.34–1.89]	0.088
		GA/GG	0.77 ± 1.25	(0.58, 0.97)	0.96 [−0.11–1.69]	
	rs10246939	CC	1.03 ± 1.42	(0.53, 1.54)	1.04 [0.35–1.87]	0.577
		TC/TT	0.88 ± 1.12	(0.72, 1.04)	0.99 [0.06–1.76]	
	WC					
*TAS1R2*	rs35874116	CC	81.32 ± 15.66	(74.85, 87.78)	78.00 [68.50–90.90]	0.632
		TC/TT	79.22 ± 13.37	(77.46, 80.98)	77.00 [68.50–88.00]	
	rs9701796	CC	78.50 ± 13.30	(76.47, 80.61)	77.00 [67.90–88.25]	0.263
		GC/GG	80.30 ± 13.26	(77.41, 83.28)	78.00 [70.25–89.00]	
*TAS2R38*	rs713598	CC	81.30 ± 13.23	(78.68, 83.99)	78.00 [70.75–90.50]	0.079
		GC/GG	78.50 ± 13.85	(76.29, 80.68)	77.00 [67.50–88.00]	
	rs1726866	AA	81.12 ± 13.30	(78.29, 83.96)	78.00 [70.00–90.50]	0.129
		GA/GG	78.38 ± 13.38	(76.32, 80.45)	77.00 [68.00–88.00]	
	rs10246939	CC	79.60 ± 13.80	(74.70, 84.50)	78.50 [71.00–86.50]	0.880
		TC/TT	79.40± 13.40	(77.60, 81.22)	77.00 [68.50–89.00]	
	WHtR					
*TAS1R2*	rs35874116	CC	0.54 ± 0.09	(0.50, 0.58)	0.51 [0.46–0.61]	0.358
		TC/TT	0.52 ± 0.09	(0.51, 0.53)	0.50 [0.46–0.57]	
	rs9701796	CC	0.52 ± 0.08	(0.50, 0.53)	0.50 [0.45–0.57]	0.227
		GC/GG	0.53 ± 0.09	(0.50, 0.55)	0.50 [0.46–0.58]	
*TAS2R38*	rs713598	CC	0.53 ± 0.09	(0.51, 0.55)	0.51 [0.47–0.59]	0.122
		GC/GG	0.52 ± 0.08	(0.50, 0.53)	0.49 [0.45–0.57]	
	rs1726866	AA	0.53 ± 0.09	(0.51, 0.55)	0.51 [0.47–0.59]	0.141
		GA/GG	0.52 ± 0.08	(0.50, 0.53)	0.49 [0.46–0.57]	
	rs10246939	CC	0.54 ± 0.09	(0.50, 0.57)	0.52 [0.47–0.59]	0.399
		TC/TT	0.52 ± 0.08	(0.51, 0.53)	0.50 [0.46–0.58]	

Nonparametric data are shown as median [25th–75th] measures of center and spread. Mann–Whitney U test was computed on anthropometric parameters. SNP: single-nucleotide polymorphism. SD: standard deviation. CI: confidence interval. *TAS1R2*: taste receptor, type1, member 2. *TAS2R38*: taste receptor, type2, member 38.

**Table 6:** Association between different genotypes with SSB consumption.

SSB Consumption/Week	Gene	Predictor	Exp (B)	95% CI		*p*-Value	Adjusted *p*-Value
				Lower	Upper		
2–3 times vs. ≤1 times	*TAS1R2*	rs35874116 CC–TC/TT	1.97	0.71	5.45	0.192	0.603
		rs9701796 CC–GC/GG	0.85	0.45	1.63	0.630	0.967
	*TAS2R38*	rs713598 CC–GC/GG	1.01	0.54	1.92	0.967	0.967
		rs1726866 AA–GA/GG	0.96	0.50	1.85	0.894	0.967
		rs10246939 CC–TC/TT	0.61	0.27	1.40	0.241	0.603
>3 times vs. ≤1 times	*TAS1R2*	rs35874116 CC–TC/TT	1.13	0.35	3.69	0.841	0.957
		rs9701796 CC–GC/GG	1.02	0.51	2.06	0.957	0.957
	*TAS2R38*	rs713598 CC–GC/GG	1.45	0.74	2.83	0.275	0.458
		rs1726866 AA–GA/GG	1.62	0.82	3.21	0.168	0.420
		rs10246939 CC–TC/TT	0.24	0.08	0.79	0.018	0.090

Combined multinomial logistic regression analysis results for each SNP (independent variable), adjusted for age, sex, nationality, BMIz scores, BMR, and PA. Mutant allele carriers, males, and non-Kuwaitis were set as the reference. The Benjamini–Hochberg correction method (FDR = 0.01) was applied to adjust the *p*-values. SNP: single-nucleotide polymorphism. Exp (B): add ratio. CI: confidence interval.

## Data Availability

Data available on request due to ethical and legal restrictions governing the use and sharing of human genetic information in Kuwait.

## Abbreviations

The following abbreviations are used in this manuscript:

WHO	World Health Organization
CDC	Centers for Disease Control and Prevention
SSB	sugar-sweetened beverages
RCT	Randomized-Controlled Trial
TAS1R2	taste receptor, type1, member 2
TAS2R38	taste receptor, type2, member 38
CD36	Cluster of Differentiation
TRPM5	Transient Receptor Potential Subfamily M Member 5
SNP	single-nucleotide polymorphism
PAV	Proline–Alanine–Valine
AVI	Alanine–Valine–Isoleucine
BMI%	body mass index-for-age percentiles
BMIz	body mass index z-scores
WC	waist circumference
WHtR	waist-to-height ratio
FFQ	Food Frequency Questionnaire
PCR	polymerase chain reaction
FDR	false discovery rate
BMR	basal metabolic rate
PA	physical activity
GWAS	genome-wide association studies
T2DM	type 2 diabetes mellitus
GLP1	glucagon-like peptide-1
CCK	cholecystokinin
MET	Metabolic Equivalent of Task
ADA	American Diabetes Association
QST	quantitative sensory testing
